# Unraveling the host range of *Plasmodium huffi*: morphological, histopathological and molecular characterization in red-legged seriemas from Brazil

**DOI:** 10.1017/S003118202500006X

**Published:** 2025-02

**Authors:** Lis Marques de Carvalho e Vieira, Sabrina Epiphanio, Natália Coelho Couto de Azevedo Fernandes, Juliana Mariotti Guerra, José Luiz Catão Dias, Maria Andreína Pacheco, Ananias A. Escalante, Érika Martins Braga

**Affiliations:** 1 Departamento de Parasitologia, Instituto de Ciências Biológicas, Universidade Federal de Minas Gerais, Belo Horizonte, MG, Brazil; 2 Departamento de Análises Clínicas e Toxicológicas, Faculdade de Ciências Farmacêuticas, Universidade de São Paulo, São Paulo, SP, Brazil; 3 Centro de Patologia, Instituto Adolfo Lutz, São Paulo, SP, Brazil; 4 Biology Department, Institute of Genomics and Evolutionary Medicine, Temple University, Philadelphia, PA, USA

**Keywords:** avian malaria, cytb, Haemosporida, histopathology, mtDNA

## Abstract

Avian *Plasmodium* parasites can be pathogenic to their vertebrate hosts. Although cases of anaemia are frequently reported in parasitized birds, the potential damage caused by the parasite during the exoerythrocytic reproduction phase remains poorly investigated. Here, we report 2 individuals of red-legged seriemas (*Cariama cristata*) infected with 2 different lineages of *Plasmodium huffi*, one of them exhibiting potential malarial-compatible tissue lesions in the spleen, liver, brain and lungs, alongside molecular confirmation of parasite presence in the spleen. Previously classified as specific to birds from the order Piciformes, this parasite has shown different associated lineages amplified across diverse host orders in South America (Anseriformes, Charadriiformes, Columbiformes, Galliformes, Pelecaniformes and Passeriformes). Those infections, however, were defined as abortive due to the absence of gametocytes visualized in blood smear slides. Herein, we confirm *P. huffi* as a generalist parasite based on the first morphological characterization in the peripheral blood of a bird outside the Piciformes order. This is also the first morphological and molecular description of a *Plasmodium* species in Cariamiformes. In addition to the morphological analyses, we have also proposed a novel phylogenetic hypothesis based on the partial *cytb* gene and the near-complete mitochondrial genome of this parasite. Our findings support that the division of the genus *Plasmodium* into subgenera is not monophyletic, as *P.* (*Huffia) huffi* and its associated lineages cluster more closely with *Plasmodium* (*Haemamoeba) gallinaceum* than with *Plasmodium* (*Huffia) elongatum*.

## Graphical abstract

**Figure:**
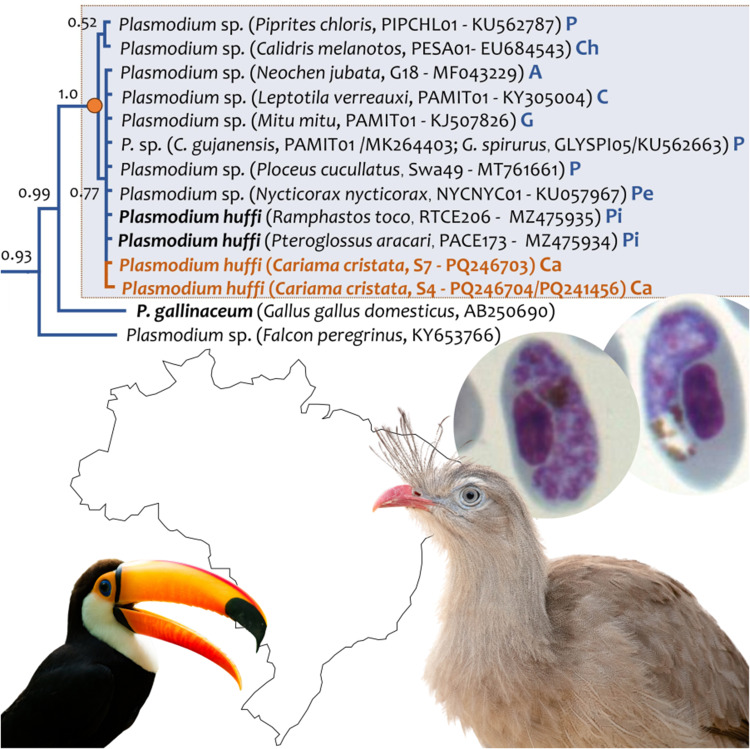

## Introduction

Haemosporidians (Apicomplexa: Haemosporida) are globally distributed parasites (Atkinson et al., 2008; Marzal, 2012; Clark et al., 2014) capable of infecting diverse classes of vertebrates (Garnham, 1966; Valkiūnas, 2005; Atkinson et al., 2008; Telford, 2009). The principal genera infecting birds, *Plasmodium, Haemoproteus* and *Leucocytozoon*, can be morphologically classified based on observable physiological processes during the erythrocytic phase (Valkiūnas, 2005). In particular, the family Plasmodiidae is characterized by the presence of haemozoin granules in their cytoplasm and the occurrence of merogony within erythrocytes. *Plasmodium* parasites are transmitted through the bites of infected culicid mosquitoes (Diptera: Culicidae) carrying sporozoites, which develop into primary meronts known as cryptozoites within lymphoid and reticuloendothelial cells of various vertebrate tissues (Bray, 1957; Huff, 1957; Garnham, 1966; Valkiūnas, 2005; Atkinson et al., 2008; Frevert et al., 2008; Telford, 2009). Only merozoites from the second generation of primary meronts (metacryptozoites) infect and multiply within erythrocytes, eventually developing into gametocytes, the infective forms for the vector. Secondary schizogony persists even during the parasitemic phase, generating meronts, which are responsible for maintaining latent infection, and potentially causing relapses (Garnham, 1966; Valkiūnas, 2005; Valkiūnas and Iezhova, 2017).

Avian Plasmodia are generalists capable of causing significant impacts on their hosts (Van Riper et al., 1986; Atkinson et al., 1995; Muriel et al., 2021). Anaemia is a commonly reported clinical condition in birds parasitized by *Plasmodium* (Valkiūnas, 2005), though increasing evidence highlights the importance of investigating potential tissue damage caused by these parasites (Coulston and Manwell, 1941; Huchzermeyer and Vyver, 1991; Atkinson et al., 2000; Valkiūnas, 2005; Ferrell et al., 2007; Palinauskas et al., 2008; Pacheco et al., 2011; Howe et al., 2012; Dinhopl et al., 2015; Ilgūnas et al., 2016; Valkiūnas and Iezhova, 2017; Pendl et al., 2022). Fatal cases have been documented in birds infected with *Plasmodium*, likely due to parenchymal organ compromise secondary to capillary blockage and endothelial damage caused by meronts (Coulston and Manwell, 1941; Huchzermeyer and Vyver, 1991; Atkinson et al., 2000; Banda et al., 2013; Vanstreels et al., 2014; Ilgūnas et al., 2016; Taunde et al., 2019; Gulliver et al., 2022). Despite this, studies including investigations of exoerythrocytic stages and their impact on the host remain limited, especially in naturally infected birds. Such analyses are essential for better understanding the pathogenesis associated with natural haemosporidian infections and the biology of these parasites.

Although the genus *Plasmodium* has been extensively studied and characterized across various avian hosts, certain bird orders remain understudied, resulting in limited knowledge about their haemoparasite communities (Lotta et al., 2019). Large-bodied cursorial birds, such as those belonging to the order Cariamiformes, are examples, given the challenges of field sampling with mist nets (Valkiūnas, 2005). This order includes 2 extant endemic South American bird species, red-legged seriema (*Cariama cristata*) and black-legged seriema (*Chunga burmeisteri*), both sedentary and commonly found in open grassland regions. The former is widely distributed across open habitats in Brazil, Uruguay, Argentina, Paraguay and Bolivia, while the latter inhabits dry forests in Bolivia, Argentina and the Paraguayan Chaco (Mayr, 2009). Seriemas are poorly studied birds susceptible to haemosporidian infections (Vanstreels et al., 2022; Vieira et al., 2023). Recent reports have identified seriemas received at wildlife rehabilitation centres as competent hosts for *Haemoproteus pulcher* (Vanstreels et al., 2022; Vieira et al., 2023), an infection seemingly common in this bird, as well as *Leucocytozoon cariamae*, the first species of *Leucocytozoon* morphologically described at lower altitudes in the Neotropics (Vieira et al., 2023). An integrative taxonomic approach has successfully identified valid species of these 2 haemosporidians, although, to date, there are no morphologically described *Plasmodium* species known to infect seriemas.

This study describes 2 lineages of the recently rediscovered species *Plasmodium huffi* in a Cariamiformes bird based on infections reported in 2 individuals of *C. cristata* in Brazil. *Plasmodium huffi* was initially suggested to be an exclusive parasite of toucans (Muniz et al., 1951; Valkiūnas, 2005; Cedrola et al., 2021), but recent findings have begun to suggest possible infections in various other avian orders through molecular data. In this study, we add *C. cristata* as a competent host for *P. huffi* based on morphological and molecular analyses. We also present a new phylogenetic hypothesis based on the parasite partial *cytb* gene and nearly complete mitochondrial DNA (mtDNA) sequences and histopathological findings that may relate to *P. huffi* infection in future studies.

## Materials and methods

### Hosts description and sampling area

Red-legged seriema is an endemic bird species of South America, occupying a vast Brazilian territory. Therefore, they are adapted to tropical climates with dry winters and humid summers, commonly found in open fields such as savannas and grasslands (Mayr, 2009). The state of Minas Gerais comprises a transition zone between the Cerrado and Atlantic Forest biomes, and seriemas can commonly be observed foraging in open fields within the Minas Gerais Cerrado (Carvalho-Roel et al., 2017).

During the study, 10 adult red-legged seriema individuals were evaluated at the Wildlife Triage Center of Belo Horizonte (CETAS-BH), Minas Gerais, Brazil, all of which were rescued in risky situations. Most had a history of being hit by vehicles in rural areas surrounding the city of Belo Horizonte. Out of 10 individuals, 2 received a microscopic positive diagnosis of *P. huffi*, specifically S4 and S7, as detailed below. One of these birds (S4) displayed clear signs of suffering and had a complete wing fracture, resulting in the decision for euthanasia. It was delivered to CETAS by a veterinary clinic that did not provide the animal’s history; hence, we do not know its origin or the cause of the fracture. A necropsy was performed on this individual.

On the other hand, the other positive individual (S7) was found in the municipality of Itabira, Minas Gerais (19° 39ʹ 57ʺ S, 43° 12ʹ 44ʺ W), approximately 100 km from Belo Horizonte. Itabira is situated in a mountainous urbanized area at approximately 795 m above sea level, with vegetation consisting of seasonal semideciduous Atlantic Forest fragments transitioning into Cerrado (Dobrzyński et al., 2021; Alvarenga et al., 2024). Following Köppen’s classification, the climate is classified as humid subtropical, with dry winters and temperate summers, averaging an annual temperature of 20.4 °C and approximately 1471 mm of precipitation (Alvares et al., 2013; Dobrzyński et al., 2021). The neighbourhood where the animal was found is considerably urbanized but surrounded by patches of forest where the individual likely inhabited. The animal’s history was not provided, but it died approximately 1 month after arrival. No necropsy was conducted in this case.

### Sampling and blood film examination

To avoid sampling parasites whose infections were acquired within the CETAS, birds had peripheral blood obtained on the same day of their arrival before being transferred to communal enclosures. Additionally, animal S4 underwent 2 further collections at 7 and 14 days post-arrival to ensure additional slides and to monitor parasitemia. Whole blood was collected by venipuncture of the brachial vein, with up to 1% of body weight volume removed. A portion was used to prepare 3 blood smear slides, and the remainder was stored in 1.5 mL microtubes containing 70% ethanol for subsequent molecular analyses.

The slides were fixed with 100% methanol for 3 mins and then stained with 10% Giemsa (pH = 7.2) for 70 mins (Valkiūnas et al., 2008). Subsequently, an Olympus CX31 microscope was used to analyse the slides at 1000× magnification. Parasitemia was quantified based on the visualization of 300 random fields, and subsequently, for comprehensive morphological analyses, all replicated slides were examined in their total extension. Parasite images were captured using an Olympus Qcolor 5 camera and processed with QCapture software. Finally, morphometric analyses were manually conducted using ImageJ software (Schneider et al., 2012) according to parameters described by Valkiūnas (2005). The nucleus displacement ratio (NDR) was calculated in accordance with Bennett and Campbell (1972).

### Organ collection and histopathology

For sample S4, the collection of the spleen, liver, brain, and lungs was conducted immediately after euthanasia. A small fragment of each organ was placed in 1.5 mL microtubes containing 70% ethanol for further DNA extraction and amplification. The remaining tissues were fixed in 10% buffered formalin and embedded in paraffin for histopathological analysis. Sections of 3 or 5 μm were obtained, stained with hematoxylin-eosin (Vanstreels et al., 2015) and Ziehl-Neelsen (Montali et al., 1976), and examined under light microscopy.

### DNA extraction, partial cytb gene amplification and sequencing

Both positive seriemas (S4 and S7) had their samples (S7’s blood and S4’s blood and organs) initially extracted at the Malaria and Parasite Genomics Laboratory of the Federal University of Minas Gerais, using the phenol–chloroform method (Sambrook et al., 1989). The samples were resuspended in 50 μL of ultrapure water and quantified using a NanoDrop 2000 spectrophotometer (Thermo Scientific, Waltham, USA®) to ensure DNA concentration in the range of 40–80 ng/μL for Polymerase Chain Reaction (PCR) amplification.

Amplification of the *cytb* fragment followed protocols described by Hellgren et al. (2004) for *Plasmodium*/*Haemoproteus*, using 1 μL of extracted DNA as a template for the first reaction and 1 μL of amplified DNA for the second reaction. Negative (MiliQ water) and positive controls (*Plasmodium falciparum*) were also included. PCR products were visualized on a 6% polyacrylamide gel stained with silver nitrate solution. In addition, to try to separate the parasite species from the sample with mixed infection (S4), the complete *cytb* gene was also amplified from 3 blood samples taken on different days and a spleen sample using the protocol reported by Pacheco et al. (2018a). Partial *cytb* gene sequences were submitted to GenBank under the accession number PQ246703 (S7) and PQ246704 (S4).

Amplified products were purified with equal volumes of sample and polyethylene 20% glycol 6000 (Sambrook and Russell, 2001), quantified using NanoDrop 2000 (Thermo Scientific, Waltham, USA®) to adjust concentration in the range of 5–20 ng/μL, and sent to the René Rachou Institute – Fiocruz/MG for bidirectional Sanger sequencing. Sequences were edited using ChromasPro 2.0.6 software (Technelysium Pty Ltd, Helensvale, Australia) and compared with databases deposited in both GenBank (http://www4.ncbi.nlm.nih.gov) and MalAvi (Bensch et al., 2009, http://mbio-serv2.mbioekol.lu.se/Malavi/).

### DNA extraction, mitochondrial genome amplification, cloning and sequencing

To get the nearly complete parasite *mtDNA* genomes of *P. huffi*, DNA was extracted from the whole blood of the sample S4 (with mixed infection and parasitamia 0·0067%) using the QIAamp DNA Micro Kit (Qiagen GmbH, Hilden, Germany). This genome was amplified using a nested PCR protocol with Takara LA Taq™ polymerase (TaKaRa Takara Mirus Bio, San Jose, USA) following Pacheco et al. (2018b), and the outer oligos forward AE170-5′ GAGGATTCTCTCCACACTTCAATTCGTACTTC 3′ and reverse AE171-5′ CAGGAAAATWATAGACCGAACCTTGGA CTC 3′, and the inner oligos forward AE176-5′ TTT CATCCTTAAATCTCGTAAC 3′ and reverse AE136-5′ GACCGAACCTTGGACTCTT 3′. PCRs were carried out in 50 μL using 5 μL of the total DNA for each PCR. Negative (distilled water) and positive controls (samples from an infected primate) were also included. Amplification conditions for both PCRs were a partial denaturation at 94 °C for 1 min and 30 cycles with 30 s at 94 °C and 7 min at 67 °C, followed by a final extension of 10 min at 72 °C. Three independent PCR products (50 μL) were excised from the gel (bands of ∼6 kb), purified using the QIAquick Gel extraction kit (Qiagen, GmbH, Hilden, Germany) and cloned into the pGEM-T Easy Vector Systems (Promega, Madison, USA) following the manufacturer’s instructions. Both strands of 7 clones were sequenced at Genewiz from Azenta Life Sciences (New Jersey, USA). All clones were identical without inconsistencies between them. The only mtDNA genome sequence obtained here was identified as *P. huffi* (new lineage) using the Basic Local Alignment Search Tool (Altschul et al., 1990) and submitted to GenBank under the accession number PQ241456.

### Phylogenetic analyses and genetic distance

Phylogenetic relationships between *P. huffi* sequences obtained in this study and previously reported sequences were estimated using both parasite partial *cytb* gene and the nearly complete parasite *mtDNA* genome. For that, 2 alignments were performed using ClustalX v2.0.12 and Muscle as implemented in SeaView v4.3.5 (Gouy et al., 2009) with manual editing. The first alignment included 54 partial *cytb* gene sequences (454 bp excluding gaps) from 4 genera (*Haemocystidium, Leucocytozoon, Haemoproteus* and *Plasmodium*) available from GenBank and the *cytb* sequences obtained here using different protocols (Hellgren et al., 2004; Pacheco et al., 2018a). All partial *cytb* gene sequences that have been reported so far as *P. huffi* or 100% identical to it (Cedrola et al., 2021) were included for comparison purposes.

A second alignment (5084 bp excluding gaps) was done using 129 mtDNA genome sequences available in the GenBank (Benson et al., 2011) for parasites belonging to 4 genera (*Haemocystidium, Leucocytozoon, Haemoproteus* and *Plasmodium*), including the mtDNA genome reported here (PQ241456). The phylogenetic analyses used sequences from *Haemocystidium, Leucocytozoon*, and *Haemoproteus* parasites as an outgroup (Pacheco and Escalante, 2023).

Then, the phylogenetic hypotheses were inferred based on these 2 alignments. Phylogenetic trees were estimated using a Bayesian method implemented in MrBayes v3.2.7 with the default priors (Ronquist and Huelsenbeck, 2003), a general time-reversible model with gamma-distributed substitution rates, and a proportion of invariant sites (GTR + Γ + I). This model was the best fit for the data with the lowest Bayesian information criterion scores, as estimated by MEGA v7.0.26 (Kumar et al., 2016). Bayesian support was inferred for the nodes in MrBayes by sampling every 1000 generations from 2 independent chains lasting 6 × 10^6^ Markov Chain Monte Carlo steps. The chains were assumed to have converged once the potential scale reduction factor value was between 1.00 and 1.02, and the average standard deviation of the posterior probability was <0·01. Once convergence was reached, 25% of the samples were discarded as a ‘burn-in’. Lineages names and GenBank accession numbers of all sequences (*cytb* and mtDNA genomes) used here are shown in both phylogenetic trees.

In addition, the average evolutionary divergences over all *P. huffi* lineage sequence pairs were estimated using the partial *cytb* gene (477 positions in the final dataset) and the Kimura 2-parameter model (Kimura, 1980) as implemented in MEGA v7.0.26 (Kumar et al., 2016). The rate variation among sites was modelled with a gamma distribution (shape parameter = 1).

## Results

### Parasite detection via microscopy and PCR

**Figure 1.**
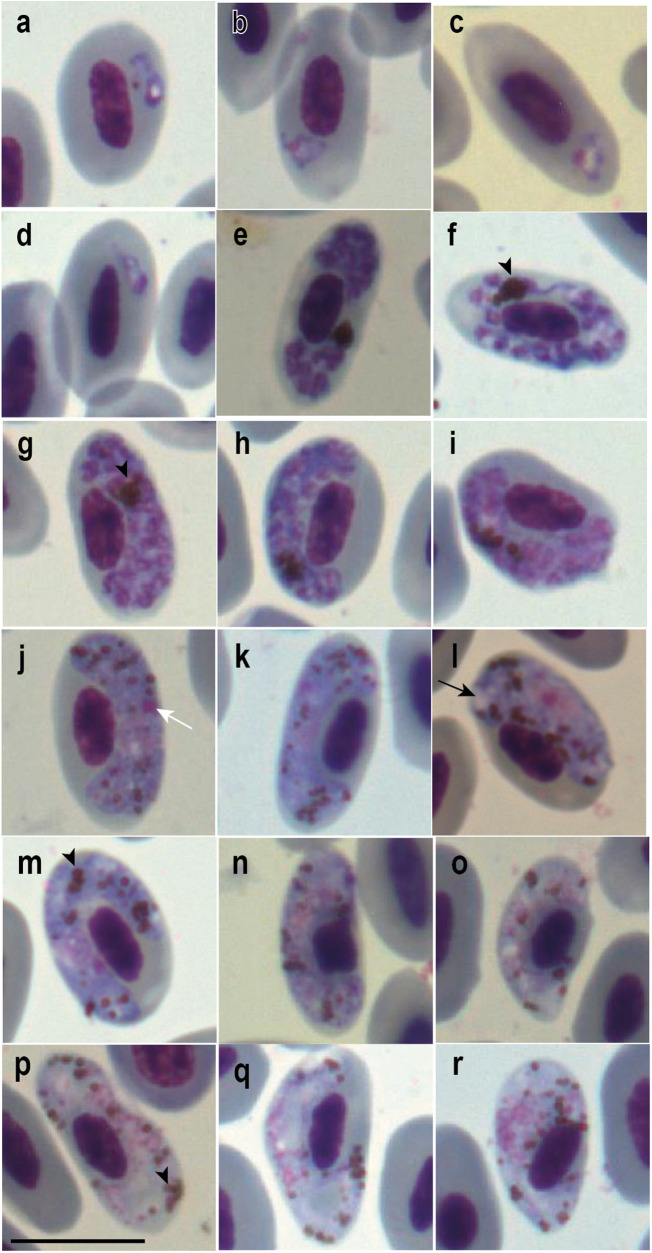
Trophozoites (a–d), meronts (e–i), macrogametocytes (j–n) and microgametocytes (o–r) of *Plasmodium huffi* from the blood of red-legged seriema (*Cariama cristata*) sampled in Minas Gerais, Brazil. Black arrowheads: haemozoin granules; black long arrows: vacuoles; white long arrows: parasite nucleolus. Giemsa-stained thin blood films. Scale bar = 10 μm.

**Figure 2.**
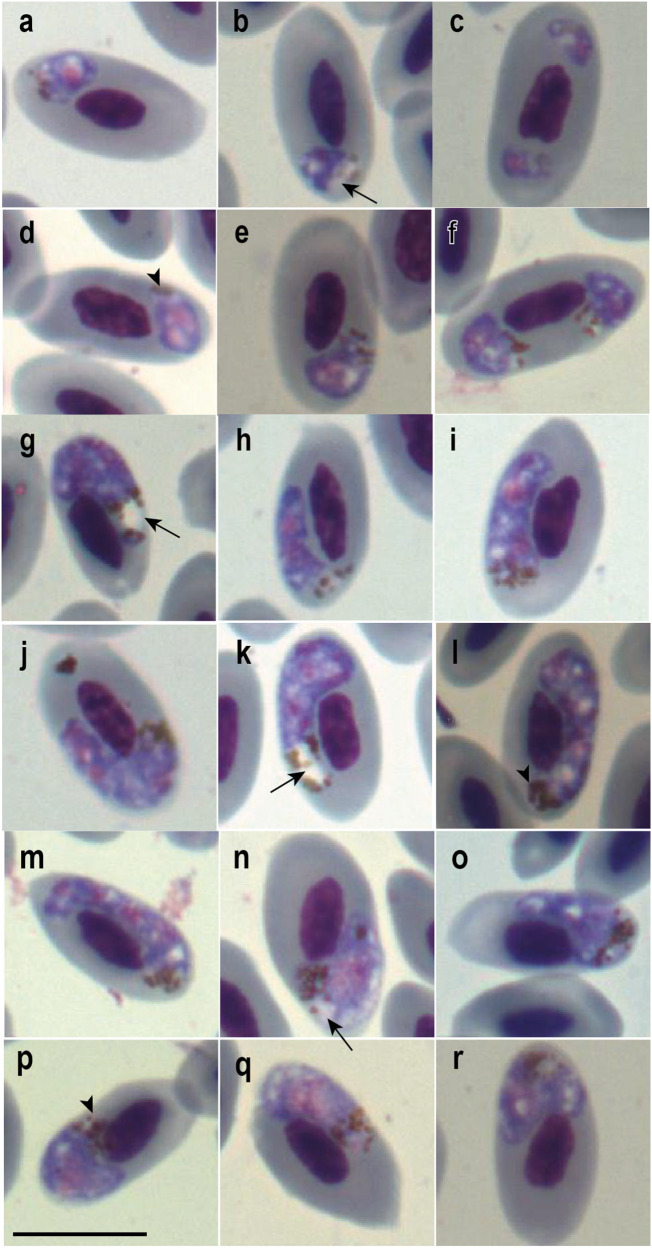
Young forms (a–f), meronts (g–m), and gametocytes (n–r) of *Plasmodium* sp. from the blood of red-legged seriema (*Cariama cristata*) sampled in Minas Gerais, Brazil. Black arrowheads: haemozoin granules; black long arrows: vacuoles. Giemsa-stained thin blood films. Scale bar = 10 μm.

Out of the 10, 2 individuals (S4 and S7) were positively diagnosed with *Plasmodium* infection by microscopy and PCR (Hellgren et al., 2004). Despite finding only 2 trophozoites on the 3 slides analysed from S7, S4 showed signs of co-infection with 2 *Plasmodium* species. This was indicated by the observation of 2 distinct morphological patterns of meronts and gametocytes on all slides, 1 resembling *P. huffi* (Figure 1) (parasitemia = 0.0067%) and the other entirely dissimilar (Figure 2) (parasitemia = 0.0167%). The morphologically distinct pattern from *P. huffi* featured parasites with a large vacuole in the polar region, where haemozoin granules frequently accumulated (Figure 2b, f, g, n, o). This is a distinctive characteristic that could be observed starting from the trophozoites and other young forms (Figure 2a–f), was highly pronounced in the meronts (Figure 2g, o), and was also present in the gametocytes (Figure 2n). This allowed us to piece together the puzzle of this second lineage and identify parasites similar to *P. huffi* for their morphometric description (Table 1). However, despite these observations on the slides, co-infection could not be confirmed using the PCR methods employed, as only 2 *P. huffi* lineages (GenBank acc. Num. S7: PQ246703 and S4: PQ246704(cytb)/PQ241456 (mt)) were amplified in several independent PCRs from these samples.

**Table 1.** Comparison of morphometric parameters of mature blood stages of *Plasmodium (Huffia) huffi* from red-legged seriema (*Cariama cristata*) and Toco toucan (*Ramphastos toco*) (Cedrola et al., 2021)

Features	***P. huffi* from *C. cristata* (S4) measurements (**μ**m)**	***P. huffi* from *R. toco* measurements (**μ**m)**
**Uninfected erythrocyte**	**(*n* = 20)**	**(*n* = 20)**
Length	11·2–13·9 (12·8 ± 0·7)	12·7–16·7 (14·3 ± 1·0)
Width	6·3–8·1 (7·1 ± 0·4)	6·6–8·6 (7·8 ± 0·6)
Area	57·4–83·0 (71·9 ± 6·6)	78·4–104·2 (6·4 ± 90·1)
Nucleus length	4·9–6·6 (6·0 ± 0·5)	7·1–8·6 (7·7 ± 0·4)
Nucleus width	2·1–3.0 (2·6 ± 0·3)	2·0–3·4 (2·6 ± 0·4)
Nucleus area	10·3–14·1 (12·5 ± 1·0)	14·2–24·2 (18·6 ± 2·7)
**Meronts**	**(*n* = 4)**	**(*n* = 6)**
Length	14·5–21·0 (16·6 ± 2·6)	15·3–18·5 (17·0 ± 1·3)
Width	3·0–3·7 (3·4 ± 0·3)	2·8–4·2 (3·4 ± 0·6)
Area	38·5–53·3 (45·3 ± 5·3)	36·9–50·9 (43·7 ± 6·7)
Number of merozoites	12·0–26·0 (16·8 ± 5·4)	12·0–28·0 (19·2 ± 6·2)
Length of infected host cell	12·2–14·3 (13·1 ± 0·9)	12·2–15·9 (14·9 ± 1·4)
Width of infected host cell	6·9–8·7 (7·8 ± 0·8)	6·9–8·2 (7·7 ± 0·4)
Area of infected host cell	75·2–86·6 (82·2 ± 4·5)	80·4–101·1 (90·9 ± 8·7)
Length of infected host cell nucleus	5·8–6·4 (6·1 ± 0·2)	7·3–13·9 (8·9 ± 2·5)
Width of infected host cell nucleus	2·8–3·4 (3·1 ± 0·2)	2·1–3·0 (2·5 ± 0·3)
Area of infected host cell nucleus	13·1–16·4 (15·2 ± 1·3)	7·0–19·6 (15·7 ± 4·8)
**Macrogametocytes**	**(*n* = 6)**	**(*n* = 23)**
Length	12·0–19·5 (16·4 ± 2·3)	17·4–25·8 (21·6 ± 2·6)
Width	3·7–5·2 (4·2 ± 0·5)	2·1–4·3 (3·3 ± 0·6)
Area	40·4–65·7 (50·7 ± 7·8)	39·2–71·5 (53·0 ± 6·5)
NDR	0·4–0·9 (0·6 ± 0·1)	0·0–0·7 (0·2 ± 0·2)
Number of haemozoin granules	20·0–32·0 (24·8 ± 3·8)	9·0–19·0 (13·4 ± 3·6)
Length of infected host cell	11·4–16·8 (13·9 ± 1·9)	16·5–27·5 (23·2 ± 1·5)
Width of infected host cell	6·1–8·0 (7·1 ± 0·6)	5·5–9·3 (6·7 ± 0·9)
Area of infected host cell	66·7–94·9 (79·8 ± 11·6)	55·6–99·9 (75·6 ± 11·1)
Length of infected host cell nucleus	3·7–6·0 (5·1 ± 0·7)	5·2–8·5 (6·5 ± 0·8)
Width of infected host cell nucleus	2·5–3·2 (2·8 ± 0·3)	2·2–3·8 (2·8 ± 0·4)
Area of infected host cell nucleus	10·1–15·1 (11·7 ± 1·7)	12·6–19·9 (15·4 ± 1·6)
**Microgametocytes**	**(*n* = 5)**	**(*n* = 11)**
Length	16·2–19·6 (18·0 ± 1·1)	16·5–30·7 (23·3 ± 3·8)
Width	3·2–5·3 (4·4 ± 0·7)	2·4–4·0 (3·3 ± 0·5)
Area	38·0–58·0 (50·5 ± 7·2)	45·5–64·9 (53·6 ± 6·7)
NDR	0·3–0·7 (0·5 ± 0·1)	0–0·8 (0·3 ± 0·3)
Number of haemozoin granules	17·0–28·0 (24·6 ± 4·2)	5·0–23·0 (16·0 ± 5·4)
Length of infected host cell	12·0–15·0 (13·6 ± 1·2)	11·4–15·8 (14·0 ± 1·2)
Width of infected host cell	6·4–7·7 (6·9 ± 0·6)	6·0–7·3 (6·9 ± 0·3)
Area of infected host cell	59·4–88·8 (75·6 ± 11·3)	64·5–88·5 (76·1 ± 8·1)
Length of infected host cell nucleus	4·8–6·5 (5·5 ± 0·6)	4·5–7·5 (6·5 ± 0·9)
Width of infected host cell nucleus	2·4–3·2 (2·8 ± 0·3)	2·2–3·5 (2·7 ± 0·4)
Area of infected host cell nucleus	10·7–15·7 (12·5 ± 1·8)	11·7–18·6 (15·3 ± 2·0)

Minimum and maximum values are provided, followed in parentheses by the arithmetic mean and standard deviation.

**Figure 3.**
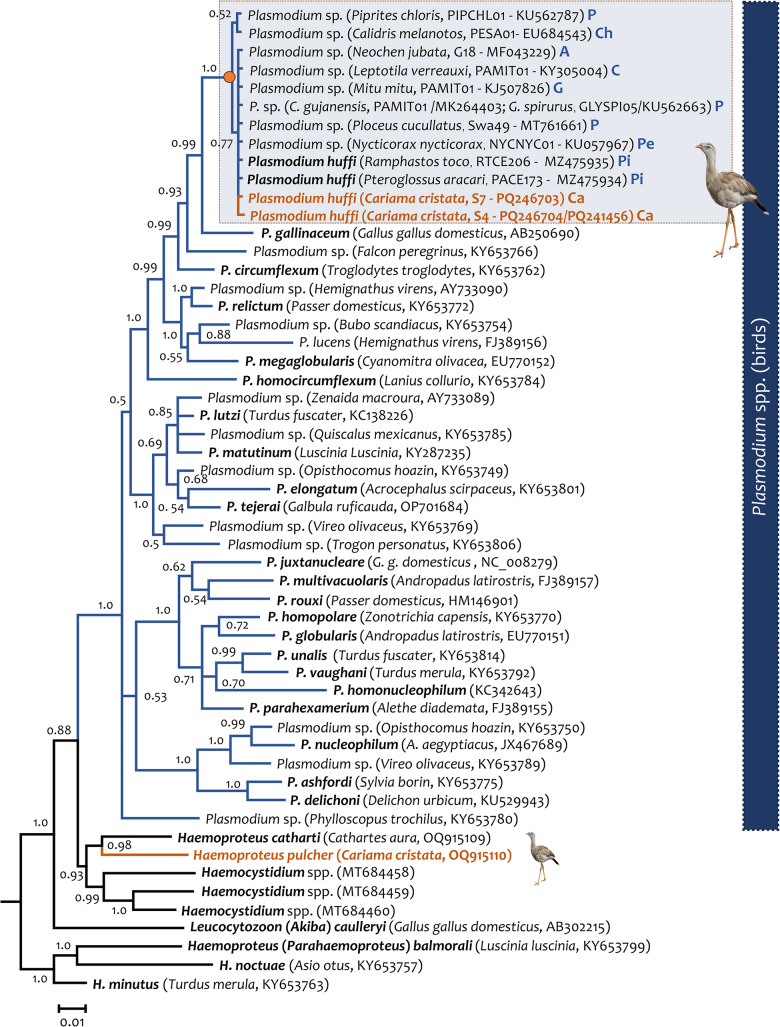
Bayesian phylogenetic hypothesis of lineages of *Plasmodium huffi* infecting red-legged seriemas (*Cariama cristata*) from Brazil based on the partial cytb gene fragment (454 bp out of the 1,134 bp of cytb gene, excluding gaps). The values above branches are posterior probabilities. The branches of the outgroup are indicated in black. Host name, GenBank accession numbers and their lineage identifiers (MalAvi database) are provided in parenthesis for the sequences used in this analysis. Plasmodium lineages recovered from seriemas are indicated in orange. A: Anseriformes; Ca: Cariamiformes; Ch: Charadriiformes; C: Columbiformes; G: Galliformes; P: Passeriformes, Pe: Pelicaniformes; Pi: Piciformes.

The sequence obtained from host S7 (GenBank acc. Num. PQ246703) showed 100% identity with the of *P. huffi* sequences RTCE206 (MZ475935) and PACE173 (MZ475934) reported by Cedrola et al. (2021), and also with strains NYCNYC01 (GenBank acc. Num. KU057967.1) found in *Nycticorax nycticorax* (Pelecaniformes) in Brazil (Chagas et al., 2016), clone G18 (GenBank acc. Num. MF043229.1) found in *Neochen jubata* in Brazil (Anseriformes) (unpublished data), and haplotype Swa49 (GenBank acc. Num. MT761661.1) found in *Ploceus cucullatus* (Passeriformes) in Eswatini (Ganser et al., 2020). On the other hand, sequencing of sample S4 (GenBank acc. Num. PQ246704 (cyb)/PQ241456 (mt)) recovered a novel lineage differing by only 1 bp (a synonymous substitution) from the lineage observed in S7. Lineages PESA01 from *Calidris melanotos* (EU684543) and PIPCHL01 from *Piprites chloris* (KU562787) differed by only 1 bp (a synonymous substitution) from the rest of the sequences (see Figure 3 and Table 2).

**Table 2.** Estimates of evolutionary divergence between different putative lineages of *Plasmodium huffi*. Standard error estimate(s) are shown above the diagonal. Analyses were conducted using the Kimura 2-parameter model [1]. The rate variation among sites was modelled with a gamma distribution (shape parameter = 1). There were a total of 477 positions in the final dataset. Evolutionary analyses were conducted in MEGA7 [2]

		Genetic distance (Standard error)								
	Parasites species (Host, lineage, NCBI code)	1	2	3	4	5	6	7	8	9
**1**	***Plasmodium huffi* (*Ramphastos toco*, RTCE206 – MZ475935)**		0·000	0·000	0·000	0·000	0·000	0·002	0·002	0·002
**2**	***Plasmodium huffi* (*Pteroglossus aracari*, PACE173 – MZ475934)**	0·000		0·000	0·000	0·000	0·000	0·002	0·002	0·002
**3**	*Plasmodium* sp. (*Nycticorax nycticorax*, NYCNYC01 – KU057967)	0·000	0·000		0·000	0·000	0·000	0·002	0·002	0·002
**4**	*Plasmodium* sp. (*Glyphorynchus spirurus*, GLYSPI05 – KU562663)	0·000	0·000	0·000		0·000	0·000	0·002	0·002	0·002
**5**	*Plasmodium* sp. (*Cyclarhis gujanensis*, PAMIT01 – MK264403)	0·000	0·000	0·000	0·000		0·000	0·002	0·002	0·002
**6**	*Plasmodium* sp. (*Neochen jubata*, G18 – MF043229)	0·000	0·000	0·000	0·000	0·000		0·002	0·002	0·002
**7**	***Plasmodium* *huffi* (*Cariama cristata*, S4 - PQ246704)**	**0·002**	**0·002**	**0·002**	**0·002**	**0·002**	**0·002**		0·003	0·003
**8**	*Plasmodium* sp. (*Calidris melanotos*, PESA01- EU684543)	0·002	0·002	0·002	0·002	0·002	0·002	0·004		0·000
**9**	*Plasmodium* sp. (*Piprites chloris*, PIPCHL01 – KU562787)	0·002	0·002	0·002	0·002	0·002	0·002	0·004	0·000	

### Morphological description of *Plasmodium huffi* from the blood of red-legged seriema

#### Trophozoites

Due to evidence of co-infection between 2 different *Plasmodium* species in sample S4, it would be impossible to attribute the morphology of young trophozoites to each species individually. Therefore, they will not be described in morphometric analyses. However, 2 morphological patterns in more mature trophozoites (Figures 1a–d and 2a–c) were observed. *Plasmodium huffi*-like trophozoites were found in both mature erythrocytes and polychromatic erythrocytes, and ring forms were observed (Figure 1c), as reported by Muniz and colleagues (1951), but not by Cedrola et al. (2021). Additionally, in agreement with both studies, amoeboid forms with filamentous extensions were observed (Figure 1a, b, d).

#### Erythrocytic meronts

Erythrocytic meronts exhibit the same distinctive characteristics reported in both previous *P. huffi* descriptions (Muniz et al., 1951; Cedrola et al., 2021). For instance, they are also found exclusively in mature erythrocytes; their young forms are more rounded and occupy polar regions of the erythrocyte (Figure 1e), whereas their mature forms elongate and spread throughout the lateral region of the erythrocyte, with their edges reaching the polar regions (Figure 1f–i). One form was found completely encircling the nucleus (Figure 1f), although this does not appear to be shared in this species. They feature scanty cytoplasm and numerous merozoites (on average, 17) when mature. Remarkably, they display an accumulation of haemozoin granules, forming a spot, thereby complicating the accurate quantification of individual granules. This characteristic was observed in all analysed meronts (Figure 1e–i). We also observed lateral displacement of the host cell nucleus caused by the parasite. No meronts completely pressed against the host cell nucleus were observed.

#### Macrogametocytes

As observed in previous descriptions, macrogametocytes were found exclusively in mature erythrocytes, with mature forms occupying a lateral position and partially encircling the host nucleus, but never completely. They are generally not pressed against the nucleus, although it can occur (Figure 1l). They may exhibit small vacuoles (Figure 1l) and have numerous haemozoin granules (approximately 25) (Figure 1m). The observed frequency of gametocytes containing these pigments, and the quantity of pigments appears to be considerably higher than reported in the redescription (see Table 1) but are in accordance with Muniz and colleague’s description. Contrary to Cedrola et al. (2021) findings, these granules do not concentrate on the polar and subpolar regions of the erythrocyte but rather disperse throughout the cytoplasm, potentially being more abundant in polar regions, as observed in Figure 1h. The forms found here resemble more closely the gametocyte reported in Figure 1l of the redescription article (Cedrola et al., 2021). In this study, the parasite nucleus did not exhibit the compact and well-stained appearance described in previous reports. Instead, the gametocyte nucleus appeared pale, poorly defined and challenging to delineate and measure. However, a well-defined nucleolus was observed (Figure 1j), which aligns with the findings of Cedrola et al. (2021), but contrasts with the observations of Muniz et al. (1951). Finally, consistent with the findings of both previous studies, we observed a significant lateral displacement of the host nucleus.

#### Microgametocytes

Microgametocytes exhibit the same general characteristics described in the macrogametocytes, except for dimorphic features such as nucleus size and shape, cytoplasm coloration and quantity and distribution of haemozoin granules. In this case, the well-defined nucleus is also absent, and it appears quite pale and difficult to delineate and measure. Muniz et al. (1951) found more haemozoin granules present in the microgametocytes than macrogametocytes, feature that is not observed in Cedrola’s redescription (Cedrola et al., 2021), but it is observed here (Table 1).

#### Remarks

Only the lineage found in seriema S4 (GenBank acc. Num. PQ246704 (cyb)/PQ241456 (mt)) had all evolutionary forms of the parasite observed on blood smear slides. Two different morphological patterns were observed on the slides, indicating a co-infection between 2 different *Plasmodium* lineages and/or species. However, only one of them was amplified through the PCR protocols applied. One of the morphospecies exhibited the same general characteristics observed in both previous descriptions of *P. huffi* (Muniz et al., 1951; Cedrola et al., 2021). This allowed the association of the amplified lineage with one of the morphospecies found on the blood smears. Due to the presence of mixed infection, we did not consider the description of trophozoites, which were indistinguishable between the 2 observed morphospecies. It is noteworthy that, similar to the study by Cedrola et al. (2021), we observed prominent nucleoli in the gametocytes, which Muniz et al. (1951) did not mention in the original description. However, the macrogametocytes frequently exhibited numerous dispersed haemozoin granules (approximately 25) in the cytoplasm, consistent with Muniz et al. (1951) but contrary to the redescriptive study (Cedrola et al., 2021). Contrary to both previous studies, which emphasized the presence of compact and well-stained nuclei in the gametocytes, all analysed gametocytes had nuclei that were difficult to delineate and pronounced paleness. Like the redescriptive study (Cedrola et al., 2021), circulating phanerozoites were not observed, as mentioned in the original description study (Muniz et al., 1951). The original description noted observations of the parasite’s exoerythrocytic forms in the spinal cord and spleen. Despite the amplification of parasite DNA in the spleen and observations of tissue alterations likely related to *Plasmodium* infection, no parasite forms were found.

Regarding morphometric analyses, generally, the gametocytes of parasites described in *C. cristata* are smaller, especially in length, compared to the gametocytes described in *Ramphastos toco*. However, uninfected erythrocytes of seriemas also appeared smaller than those of uninfected Tucano erythrocytes (see Table 1).

### Phylogenetic analyses

**Figure 4.**
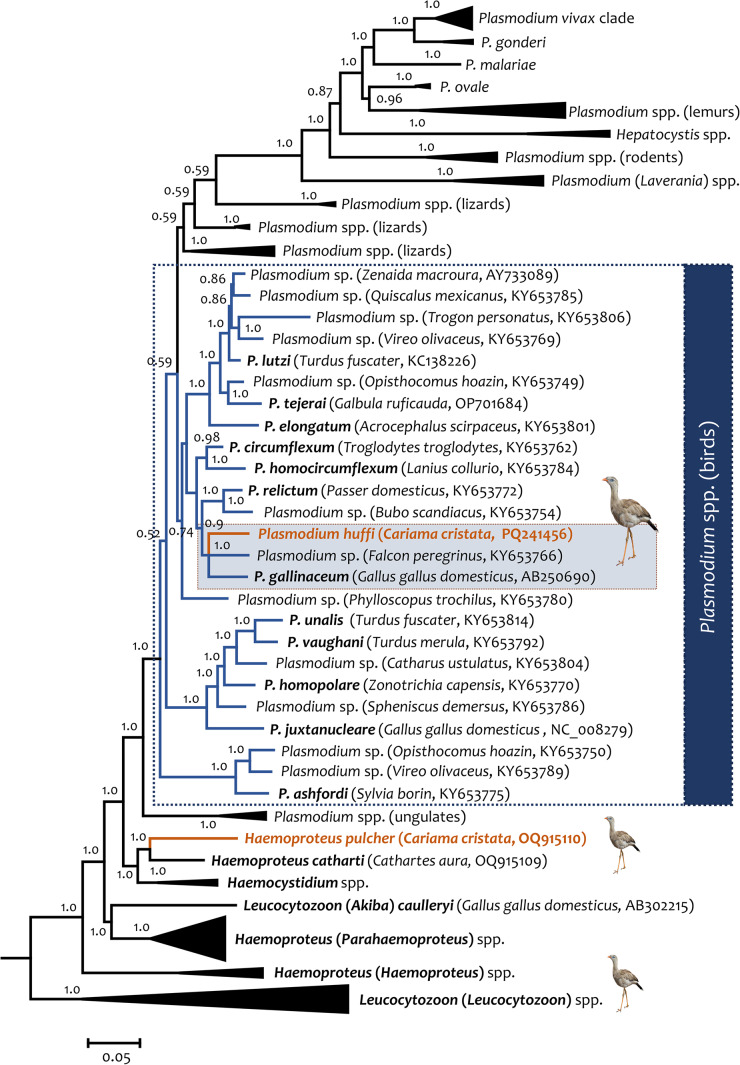
Bayesian phylogenetic hypothesis of *Plasmodium huffi* infecting red-legged seriemas (*Cariama cristata*) from Brazil based on the mitochondrial genome (5084 bp excluding gaps). The values above branches are posterior probabilities. the branches of the outgroup are indicated in black. Host names and GenBank accession numbers are provided in parentheses for the sequences used in this analysis. *Plasmodium* lineages recovered from seriemas are indicated in orange.

Phylogenetic relationships between the 2 *Plasmodium* lineages found in this study (S4 and S7) and the *P. huffi* (PACE173 and RTCE206) partial *cytb* gene sequences reported in toucans (Piciformes) are shown in Figure 3. As expected, these *cytb* sequences clustered with some morphologically uncharacterized parasite sequences isolated from other 6 avian orders (Galliformes, Anseriformes, Pelecaniformes, Charadriiformes, Columbiformes and Passeriformes). In both phylogenetic trees, estimated with the partial cytb gene (Figure 3) and the mtDNA genome (Figure 4), *P.* (*Huffia*) *huffi* appeared sharing a common ancestor with *Plasmodium* (*Haemamoeba*) *gallinaceum* from *Gallus gallus domesticus* (lineage GALLUS01, AB250690) and *Plasmodium* sp. from a *Falcon peregrinus* (KY653766). It is worth noting that the phylogenetic relationship of *Haemoproteus pulcher* found also in red-legged seriemas (OQ915110) was also confirmed here including more *cytb* and mt genomes sequences.

### Histopathology of S4 sample

**Figure 5.**
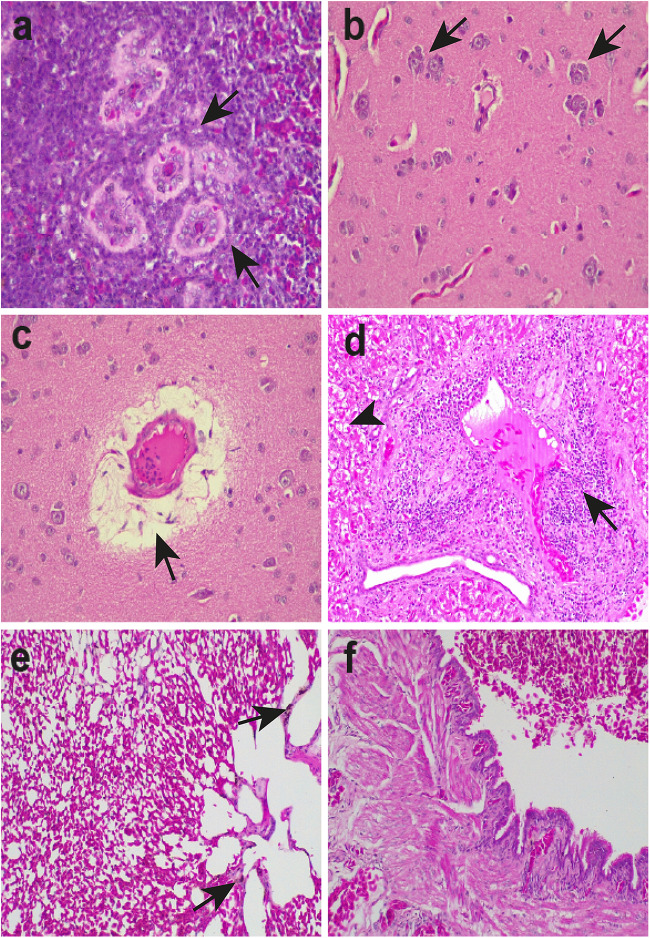
Histological sections of spleen (a), brain (b, c), liver (d) and lungs (e, f) of S4 red-legged seriema (*Cariama cristata*) infected with *Plasmodium huffi* (a, d, g). (a) Spleen (400×): hyperplasia/trophy of perivascular dendritic reticular cells (arrows), (b) brain (400×): satelitosis (arrows) and congestion (head arrow), (c) brain (400×): cerebral oedema (arrow), (d) liver (200×): periportal lymphoplasmacytic hepatitis with the presence of heterophils (arrow). Micro and macrovacuolar degeneration with hepatocellular oedema (head arrow), (e) lung (200×): moderate congestion and haemorrhagic areas with the presence of hemosiderosis (arrow) and (f) lung (200×): haemorrhage in the mesobronchial region.

In the spleen of the S4 individual, there was hyperplasia and hypertrophy of perivascular dendritic reticular cells, lymphocytolysis, red pulp congestion, and hemosiderosis (Figure 5a). In the brain, there was evidence of perivascular oedema, congestion and satellitosis, characterized by glial cell reaction around neurons (Figure 5b, c, e). The liver exhibited hepatocellular micro- and macro-vacuolar degeneration with cellular oedema. Periportal lymphoplasmacytic hepatitis was noted with heterophil presence, as well as nests of distended vacuolated cells surrounded by inflammatory infiltrate in the periportal structure (Figure 5d). Mild congestion and hemosiderosis were also present in the organ. Finally, the lungs showed moderate congestion and haemorrhagic areas with hemosiderosis, mesobronchial haemorrhage and anthracosis (Figure 5e, f). The presence of acid-fast bacilli, such as *Mycobacterium* spp., was ruled out in all samples through Ziehl-Neelsen staining. Mycobacteriosis is frequently reported in both domestic and wild birds of various orders (Dhama et al., 2011; Shivaprasad and Palmieri, 2012) and commonly induces granulomatous lesions in multiple organs (Montali et al., 1976; Shivaprasad and Palmieri, 2012), thus justifying the differential diagnosis conducted in this study.

## Discussion

This study describes 2 lineages corresponding to the recently rediscovered species *P. huffi* in red-legged seriemas, a Cariamiformes bird. *Plasmodium huffi* infections were detected in 2 individuals of *C. cristata* received by the CETAS-BH, Brazil. This *Plasmodium* species was first described in Brazil parasitizing a Toco Toucan (*R. toco*) (Muniz et al., 1951). However, the characterization was inconclusive, leading to the parasite being considered a lost lineage or *species inquirenda* (Valkiūnas, 2005). A redescription of the species was conducted in 2021 by Cedrola and colleagues based on infections also reported in Toco Toucan (*R. toco*) and Black-necked aracari (*Pteroglossus aracari*) in Brazil. In both descriptions, *P. huffi* has been assigned to be a specialist parasite to Piciformes, even with the rising amount of lineage descriptions across various bird orders (Passeriformes, Pelecaniformes, Charadriiformes, Galliformes, Columbiformes and Anseriformes) (Yohannes et al., 2009; Lacorte et al., 2013; Chagas et al., 2016; Ferreira-Junior et al., 2017; Fecchio et al., 2018, 2021). Finding a molecular lineage without information on gametocytes does not allow determining whether a host is competent or incidental (Valkiūnas, 2005; Valkiūnas et al., 2014; Anjos et al., 2021; Cedrola et al., 2021; Pacheco et al., 2022; Valkiūnas and Iezhova, 2023). Nevertheless, the extensive range of host species where the parasite has been detected using molecular methods suggested that *P. huffi* could have a broad host range.

We confirm here the first competent infection of *P. huffi* outside the order Piciformes through molecular studies associated with morphological description. This provides evidence that, like other *Plasmodium* species, it is a generalist parasite and is potentially infective across different orders of hosts in South America. Therefore, further investment in the morphological characterization of lineages in other host orders is needed to confirm the parasite’s reproductive success in different bird species. Together with the morphological characterization, we present a novel phylogenetic hypothesis based on the partial *cytb* gene and this parasite’s nearly complete mitochondrial genome, along with histopathological analyses of various organs from one of the parasitized seriemas. Parasite DNA was successfully amplified from the spleen of 1 seriema. Histopathological examinations of the spleen, liver, brain and lungs revealed damage in all evaluated organs. While some of this damage suggests infections by *Plasmodium*, it is necessary to apply more sensitive methods to visualize the exoerythrocytic stages of the parasite before making any conclusions.

### Remarks on the lineages and hosts analysed in this study

The seriema individual S4 presented a co-infection involving 2 different *Plasmodium* lineages, with only 1 amplified by the PCR methods (*P. huffi*). This lineage is novel and differs by 1 bp (a synonymous substitution) from RTCE206 (GenBank acc. Num. MZ475935.1) found in *R. toco* and PACE173 (GenBank acc. Num. MZ475934.1) found in *P. aracari*. The co-infection was identifiable on the blood slides, which revealed 2 morphologically distinct forms. One morphotype closely resembled *P. huffi* (Figure 1), while the other exhibited unique characteristics across various developmental stages, including young forms, meronts and gametocytes (Figure 2). This enabled morphological distinction and confirmed host competence for this *Plasmodium* sp. lineage. However, the sequence lineage found in the S7 individual is 100% identical to the previous *P. huffi* sequences reported in toucans (Cedrola et al., 2021).

The unsuccessful obtention of one of the *Plasmodium* lineages reinforces the need for new molecular protocols that use next-generation sequencing (Pacheco et al., 2024) to recover both lineages present in a mixed infection. Mixed infections by haemosporidians are commonly found in free-living birds, and widely used PCR protocols targeting the *cytb* gene are known to underestimate these infections (Pérez-Tris and Bensch, 2005; Bernotienė et al., 2016; Pacheco et al., 2018a, 2024). Again, we emphasize the necessity of meticulous analysis of blood smears, as although it is a low-sensitivity method, it can be used to effectively visualize different infection patterns and detect possible mixed infections that are underestimated by the most common molecular methods (Pacheco et al., 2022; Valkiūnas and Iezhova, 2023; Vieira et al., 2023).

Interestingly, what appeared to be a parasite species restricted to South America was also reported in Village Weaver (*Ploceus cucullatus*, Ploceidae), a non-migratory passerine from Southern Africa (Ganser et al., 2020). This finding necessitates further studies on the geographic distribution of this species, confirmation of competent hosts outside Brazil, and phylogeographic analyses to determine if *P. huffi* is spreading from Brazil, where it was initially identified and where the majority of lineages are reported (Muniz et al., 1951; Valkiūnas, 2005; Lacorte et al., 2013; Chagas et al., 2016; Ferreira-Junior et al., 2017; Fecchio et al., 2018, 2021; Cedrola et al., 2021).

### *Plasmodium huffi* morphological remarks

The original description of *P. huffi*, conducted in 1951 by Muniz and colleagues on toucans (*R. toco*) at the Zoological Garden of Rio de Janeiro, Brazil, presented inconsistencies. This was due to the type of material showing co-infection between *P. huffi* and another *Plasmodium* species of the subgenus *Novyella*, leading to an insufficient characterization of the parasite (Huff, 1953; Garnham, 1966). In 2021, a study molecularly detecting the same lineage in the same host with sufficient parasitemia for morphological characterization successfully recovered the lost lineage and fully characterized it (Cedrola et al., 2021). The morphological characteristics in both studies are similar, and the fact that the species was found in the same host species may have further contributed to their similarity (Laird and Van Riper, 1981; Valkiūnas, 2005; Vanstreels et al., 2014).

In this study, some morphological differences can be observed compared to previous descriptions, particularly regarding the gametocytes. The meronts are very similar, showing numerous merozoites and the characteristic spot of haemozoin granules in the cytoplasm (Figure 1e–i). However, the gametocytes presented here differ primarily due to a higher occurrence and quantity of haemozoin granules dispersed throughout the cytoplasm, and the paleness of the parasite nucleus (Figure 1j–r). Despite the 1951 description stating the presence of numerous haemozoin granules (on average, 17) dispersed in the cytoplasm, which is still below what we observed (Table 1), the images in the redescriptive study show even fewer granules (see Figure 1 in Cedrola et al., 2021). Although their calculated average was 13.4 granules for macrogametocytes and 16 for microgametocytes, only 1 gametocyte in the image plate (see Figure 1l in Cedrola et al., 2021) presents haemozoin granules. Additionally, both the 1951 description and the 2021 redescriptive study characterized the nucleus as ‘compact and brightly stained’, which was not observed in any individuals shown here. These inter-population variations are common in haemosporidian studies and can arise from differences in host factors that might differentially shape some physiological characters of the parasite (Laird and Van Riper, 1981; Valkiūnas, 2005; Vanstreels et al., 2014; Vieira et al., 2023). Besides having been observed in wild animals, this has also been demonstrated in experimental infections. An example is *Plasmodium subpraecox*, which developed morphologically distinct blood forms in terms of size, shape, number of merozoites, quantity and distribution of haemozoin granules when infecting canaries and owls (Garnham, 1966; Valkiūnas, 2005), birds from phylogenetically distant orders, which is also true for the orders Cariamiformes and Piciformes (Prum et al., 2015). As discussed by Valkiūnas (2005), these variations are evolutionarily shaped, and the differences observed were possibly due to a recent adaptation of the parasite to owls, which has not yet reached stability. Differences in sample preparation can also influence the visualization of certain features, such as volutin granules (Valkiūnas, 2005; Ferreira-Junior et al., 2018; Vieira et al., 2023). Despite using Giemsa staining in both studies, variations in staining intensity due to differences in dye brand, dilution process, water pH and staining duration could result in distinct staining outcomes (Horobin and Walter, 1987; Stefanović et al., 2013; Stockert et al., 2014), which would explain the less stained nucleus observed in the material presented here. Despite variations in gametocytes, a prominent nucleolus at the margin of the parasite nucleus was observed, as described by Cedrola et al. (2021), a characteristic not noted in the original description (Muniz et al., 1951).

Although trophozoite characterization was insufficient in this study due to the present mixed infection, the few patterns observed here that we could attribute to *P. huffi* were consistent with both previous description studies (Figure 1a–d).

### *Plasmodium huffi* molecular remarks

The phylogenetic hypothesis, derived from the partial *cytb* gene and nearly complete mitochondrial genome, supports previous phylogenies based only on the partial *cytb* gene marker. The lineages associated with *P. huffi* continue to group more closely with *P.* (*Haemamoeba*) *gallinaceum* than with *Plasmodium* (*Huffia) elongatum*. This reinforces the idea that the division of the *Plasmodium* genus into different subgenera based on morphological characteristics is not monophyletic. Instead, it reflects a series of homoplastic characters (Martinsen et al., 2008; Hernández-Lara et al., 2018; Cedrola et al., 2021).

### Histopathological remarks

Analyses performed on toucans (*R. toco*), both naturally and experimentally infected with *P. huffi*, indicated numerous meronts in the bone marrow and spleen (Muniz et al., 1951; Valkiūnas, 2005). Indeed, in this study, molecular analyses conducted on fragments from the lungs, brain, liver and spleen of one of the seriemas examined detected the parasite’s DNA only in the spleen. Nevertheless, no exoerythrocytic stages of *P. huffi* were found in the histopathological analyses, suggesting that we cannot directly link the observed lesions to *Plasmodium* infection. It is important to highlight that the detection of meronts in lesions during histopathological studies is quite rare (Silveira et al., 2013; Vanstreels et al., 2014), especially without employing more sensitive techniques such as chromogenic in situ hybridization (CISH) (Dinhopl et al., 2010; Himmel et al., 2019). However, it is worth mentioning that, despite its sensitivity, this technique presents limitations in detecting exoerythrocytic stages. In a recent study conducted by Valkiūnas et al. (2024), the researchers examined dozens to hundreds of tissue sections from 7 birds infected with *Haemoproteus*. Despite the high levels of parasitemia observed in all the individuals, no tissue stages of the parasite were found in any of them. Therefore, it is crucial for the samples obtained in this study to undergo CISH analysis to accurately determine the presence or absence of *P. huffi* tissue stages in the analysed seriema. This analysis would enhance our understanding of the exoerythrocytic cycle of the parasite and the potential damage it may cause.

Although we cannot directly link the observed histopathological changes to the malarial infection in this case – mainly since the animal is a wild species with no previous medical history – some of the damage has been documented in other birds infected with *Plasmodium* (Figure 5). For example, hepatic and splenic hemosiderosis are characteristic processes of avian malaria (Valkiūnas, 2005; Taunde et al., 2019) and have been reported in naturally infected captive birds, such as little penguins (*Eudyptula minor*) and kiwis (*Apteryx mantelli*) infected with *P. elongatum* in New Zealand (Banda et al., 2013; Sijbranda et al., 2017; Gulliver et al., 2022), Humboldt penguins (*Spheniscus humboldti*) infected with *Plasmodium matutinum* in the United Kingdom (González-Olvera et al., 2022), among other examples (Howe et al., 2012; Vanstreels et al., 2015; Verwey et al., 2018; Taunde et al., 2019). The presence of hepatitis is also reported (Ferrell et al., 2007; Howe et al., 2012; Vanstreels et al., 2015), with periportal lymphoplasmacytic hepatitis already described in captive masked bobwhite quails (*Colinus virginianus ridgwayi*), possibly associated with infection by a *Plasmodium juxtanucleare*-like parasite (Pacheco et al., 2011). Processes of hepatosplenic (Gulliver et al., 2022) and pulmonary congestion (Banda et al., 2013; Sijbranda et al., 2017) are also common, as are hypertrophic and hyperplastic processes in different organs (Bak et al., 1984; Palinauskas et al., 2008; Gulliver et al., 2022), observed here only in perivascular dendritic reticular cells of the spleen (Figure 5a). The process of lymphocytolysis observed in the spleen (Figure 5a) has also been seen in Magellanic penguins (*Spheniscus magellanicus*) infected with different *Plasmodium* species and lineages (Vanstreels et al., 2015).

Many causes of death due to avian malaria are associated with the failure of parenchymal organs secondary to the proliferation of meronts in endothelial cells of various tissues, as the lungs (Vanstreels et al., 2014; Ilgūnas et al., 2016; Gulliver et al., 2022). Thus, a frequent histopathological finding is the presence of interstitial pneumonia (Atkinson et al., 2000; Pacheco et al., 2011; Howe et al., 2012; Banda et al., 2013; Vanstreels et al., 2014, 2015; Sijbranda et al., 2017; González-Olvera et al., 2022; Gulliver et al., 2022), which was not observed in the studied seriema. However, the lungs exhibited haemorrhagic features (Figure 5e–f), a phenomenon uncommon in *Plasmodium* infection and that could be related to mechanical trauma. Only a few studies report cerebral alterations, such as the presence of fibrin thrombi in capillaries (Silva et al., 2021; Gulliver et al., 2022). The presence of oedema, congestion and satellitosis (as shown in Figure 5b–c), which is characterized by a glial cell reaction surrounding neurons, may result from several processes, including degeneration, hypoxia and inflammation. These changes could be linked to parasitic infection, but they are largely nonspecific.

## Conclusions

Further elucidation of *P. huffi*’s host diversity is crucial for a comprehensive understanding of transmission dynamics and biogeography of this parasite, which is now proven to be generalist. While avian Plasmodia are widely studied and their generalist biology is well-recognized for most described species (Valkiūnas, 2005), classifying a parasite as host-specific based on limited sampling and laboratory experimental infections studies can lead to premature or potentially incorrect conclusions. *Plasmodium huffi* has shown potential pathogenicity related to its exoerythrocytic cycle in a *C. cristata* individual; however, further research is necessary to effectively connect tissue stages to histopathological changes. Seriemas have emerged as significant hosts for haemosporidians in Brazil. Over the past 2 years, our research group has contributed to the morphological and molecular descriptions of species of *Leucocytozoon, Haemoproteus* (Vieira et al., 2023), and, in this study, documented the first instance of *Plasmodium* infecting Cariamiformes using molecular and morphological characterizations. This calls for increased sampling efforts to better understand the protozoan community of historically understudied birds, such as those belonging to the order Cariamiformes.

## Data Availability

The *mtDNA* genome sequence and the *cytb* partial sequences obtained in this study are deposited on GenBank under accession numbers PQ241456, PQ246703 (S7) and PQ246704 (S4).
